# On Hydrocephalic Affection

**Published:** 1822-03

**Authors:** 


THE LONDON
### Medical and Physical Journal.

3 OF VOL. XLVII.] MARCH, 1822. [No. 277.

For many fortunate discoveries in medicine, and for the detection of numerous errors, the world is indebted to the rapid circulation of Monthly Journals; and there never existed any work, to which the Faculty, in Europe and America, were under deeper obligations, than to the Medical and Physical Journal of London, now forming a long, but an invaluable, series.—RUSH.

### Original Communications, Select Observations, etc.

### PATHOLOGY.

ART. I.—*On Hydrocephalic Affection.* By Dr. KINGLAKE.

HYDROCEPHALIC affection would appear to be to the brain very much what hydrothorax and ascites are to the thoracic and abdominal viscera. In these different situations, a serous effusion has alike taken place, arising either from redundant exhalation or deficient absorption. The analogy of these affections farther holds, in being rather morbid effects than original or idiopathic ailments. Redundant and stagnant exhalations in interstitial cavities do not, in the first instance, occur; but are, perhaps, invariably the result of much previous disorder, by which the various evils connected with an unequal distribution of vital action, and of the circulating fluids, have been incurred.

Either undue vascular distention, or excitement, in any situation, will occasion diseased action; and, when it occurs in a visceral organ, whether of the cranial, thoracic, abdominal, or pelvic cavities, it must induce a high degree of irritation, tending to awaken dormant sympathies, and to distemper, more or less, the general excitability of the system.

Dropsical affections, of which hydrocephalus is a variety, are usually the ultimate effect of both acute and chronic diseases. In whatever part of the animal system morbid action originates, it may progressively become so disseminated and complicated, as to assume a general and an inveterate character. Contusions, wounds, tumors, and all the external causes of inflammatory excitement, may so disturb the healthy state of vital action, as to occasion, by sympathetic or associative influence, serious ailment, in either the contiguous or remote viscera, according to existing circumstances of greater or less susceptibility for being morbidly affected. Tendinous and ligamentous excitement may so distemper the vital action of the whole system, as to induce visceral disease, particularly of the first passages and brain. Whatever powerfully disorders the action of the heart and arteries, may derange the various secretions, and all the vital processes on which life and health depend. In tracing the causes of dropsical disease, reference must be chiefly had to the digestive organs, and to the disabling, and often irreparable, effects of protracted fever, and other painful and debilitating affections.

The strong sympathetic influence subsisting between the abdominal and thoracic viscera, and, indeed, in a considerable degree, between every part of the system, with the brain, subjects that organ to inordinate impressions from the diseases of these parts, especially when painful and of long duration. The way in which sympathetic influence is extended to the brain, in common with all other instances of transferred or associated disease, is by causing an increased excitement, and an additional flow of circulating fluids, on that viscus. The effect of this determination, and consequent increased exertion of vital power on the brain, would be to render it morbidly excitable, and to load its sanguiferous vessels with a larger quantity of fluid than could be freely transmitted through them. The direct result of this state would be excessive vascular distention, diminishing the contractile power of the vessels so distended, and inducing impeded circulation, congestive fullness, and painful oppression. This morbid condition cannot long endure, without causing the exhalants of the brain to be surcharged with fluids, from whence the ventricular cavities, and the interstices of the convolutions of that organ, may be deluged with serous effusion or sanguineous extravasation. Although this dropsical effusion commonly arises from an increased action of the exhalants, yet a similar effect may proceed from atony and torpor of these vessels, induced by diminished vital power, and the almost-paralyzing exhaustion of chronic disease.

Stomachic and duodenal oppression, from indigestion, and a vitiated secretion of the gastric, enteric, and biliary fluids, with mesenteric obstruction and constipated bowels, have, conjointly and separately, a strong tendency to destroy, by sympathetic agency, the healthful state of the brain, both in respect of its nervous function, and in that of its regularly transmitting through its various structures its proportion of the circulating fluids. Fevers of all descriptions, whether actively or passively inflammatory,—whether distinctly originating from visceral derangement, or, more obscurely, from a distempered state of vital power, pervading, more or less, the whole system, inducing unnatural proneness or susceptibility for diseased impressions,—may deposit and establish on the brain destructive pressure. Febrile affections, of whatever type, are apt, by long continuance, to assume the typhoid character, or that condition of disease in which venous plethora and visceral congestion oppress and endanger the extinction of the vital power. These cases are often formidable, and always of precarious issue, by involving the brain in the serious difficulties attending watery effusions.

Morbid determinations to the brain, arising from diseases situated in other parts of the frame, produce various effects, according to the temperament of the patient, and more particularly as that organ may chance to have its vital energies more or less diminished or depressed by the noxious influence. When the pressure goes far enough to induce violent excitement, and is not sufficient to occasion paralyzing torpor, the symptoms will exhibit a corresponding character. In the one case excessive, in the other deficient, excitement, will prevail. Thus, contracted states, in the several forms of phrensical and lethargic affections, may result from different degrees of the same offensive pressure. Hydrocephalic malady, therefore, may be regarded as one of the most fatal sequelæ of other diseases. It cannot justly be considered as a primary affection, but may, almost invariably, be held to be of a secondary nature, originating in disordered actions in other parts of the frame, and extended to the brain by sympathetic influence.

The organic arrangement and function of the brain are of a nature to secure it, for the most part, from initiating diseases. The natural processes of that organ are of the most measured and regulated kind. The cerebral texture is too tender and yielding to admit of violent exertions of vital power, compatibly with either health or life. The diseases of the brain, therefore, are, in most instances, produced by the sympathetic influence of ailments in other situations, rather than from any immediate defect in its own healthy condition. This may be regarded as the case very generally, and more particularly in the dropsical affection to which the cerebral organ, in common with all other visceral and cellular tissues, is liable.

Thoracic, abdominal, ovarial, scrotal, and anasarcous, like cerebral dropsies, are not original diseases, but result from the active progress of other disorders; and denote, in every instance, a redundant exhalation, or a larger deposit of lubricating fluids on the interstitial surfaces than can be removed by the absorbing vessels of the inundated part, and passed on in the course of the lymphatic circulation. The brain is the main organ in which sensibility is generated; the vital processes, obtaining in its structure, produce sensorial power; it cannot, therefore, fail to be pre-eminently sentient. This property renders it liable to partake, readily, in the various diseases incident to animal life. It enjoys, from situation and organization, important immunities from original disease; but its vital connexion is so interwoven and identified with every part of the system, as to subject it to a risk of eventually sharing, in a greater or less degree, in every variety of morbid derangement that may violently and lastingly disorder the health of other parts.

It is of great practical moment duly to consider that hydrocephalic affection is an effect of other diseases, and that it does not directly arise from any defect in the parts containing the watery effusion. Early and assiduous attention should be paid to the disease which may threaten an ultimate production of dropsy of the brain. When the leading symptoms, denoting aqueous oppression of the cerebral organ, such as impaired vision, dull hearing, unsteady muscular motion, vertiginous sensations, mental fatuity, insanity, &c. occur, the prospect of affording any relief is very distant. In the very nature of the undertaking, it is attempting an almost unattainable object. This consideration shows that there should be no delay in employing the most active and promising remedies. Hydrocephalic affection, however unfavourably circumstanced, should not be neglected, as it is impossible, from appearances, accurately to distinguish whether the condition of the disease be merely sympathetic, or that of a positive establishment of its mischief, independently of distant influence.

The sympathetic diseases of the brain are less dangerous at the commencement than those which originate in the organ itself; yet but a short continuance of the morbid determination of excitement and vascular fullness, eventually terminating in serous effusion into the ventricles, will be sufficient to render it quite independent of the cause that may have produced it. Fits of indigestion are often accompanied by a state of cerebral affection, that assumes a very imposing aspect of original and deadly malady. Undigested fruit, particularly nuts and plums, also shell-fish, &c. have been occasionally attended with such serious affection of the brain, as to have caused a suspension of the higher mental and nervous functions of that organ; all which has subsided on obtaining copious evacuations from the bowels. Without being ever able to ensure the brain an exemption from sympathetic disease, even to the extent of dropsical effusion, vascular depletion by bleeding, and full and free discharges from the intestines, should not be omitted. The length to which these leading remedies should be carried, can only be determined by the effect produced, and the state of vital power for enduring them. They should be sedulously employed, either until relief be obtained, or the faultering remains of life may forbid further exhaustion. Active diuretics, as in other instances of dropsy, and blistering the lower extremities, on the principle of derivation, should be tried. In the advanced stages of hydrocephalic disease, saturating the system with mercurial influence, either by inunction or by giving the submuriate of quicksilver, in doses of five grains every four hours, would be advisable. If the peristaltic power of the intestines be torpid, its active excitement will be strongly indicated, both in a derivent consideration, and with a view to obtain the increased secretion and discharges from the bowels that are likely to be salutary in cerebral affections.

The hydrocephalic ailment, by which children are often so deplorably cut off, is of the most insidious description. Indigestion from dietetic indulgences, and constipated bowels, by which the first passages are oppressed and otherwise disordered, the biliary and enteric secretions become irregular and vitiated, chylification is rendered imperfect, and the mesenteric glands are distempered,—most commonly occasion it. These ailments often prove fatal, not in the original seat of disease, but in their sympathetic or transferred position in the brain. Abstaining from fermented liquors of every kind, and subsisting chiefly on vegetable diet, with an habitually soluble state of the bowels, would rescue multitudes of children from hydrocephalic destruction. Restraining and subduing febrile disease, by suitable bleedings and other evacuations, at an early period, would, also, happily alleviate the mortal havoc that is made of human life, by the protracted duration of fever finally casting on the brain destructive oppression and exhaustion. Diseases of every description should, if possible, be overcome in the situations in which they first arise, lest, by continuance, they should at once acquire additional force and malignity, and be at length transferred, by intermediate sympathy, to the brain, where such transposition may produce various kinds and degrees of affliction; and where it very often actually occasions hydrocephalic extinction of life.

*Taunton; November 13th,* 1821.

---

ART. II.—*Case of Sanguineous Apoplexy occurring in a Child.* By T. H. GREENHOW, Esq.

